# A Novel Method for Objective Selection of Information Sources Using Multi-Kernel SVM and Local Scaling

**DOI:** 10.3390/s20143919

**Published:** 2020-07-14

**Authors:** Henry Jhoán Areiza-Laverde, Andrés Eduardo Castro-Ospina, María Liliana Hernández, Gloria M. Díaz

**Affiliations:** 1 MIRP Lab–Parque i, Instituto Tecnológico Metropolitano (ITM), Medellín 050013, Colombia; henryareiza135582@correo.itm.edu.co (H.J.A.-L.); andrescastro@itm.edu.co (A.E.C.-O.); 2 Grupo de Investigación del Instituto de Alta Tecnología Médica (IATM), Ayudas Diagnósticas Sura, Medellín 050026, Colombia; mlhernandezp@sura.com.co

**Keywords:** machine learning, multimodality, multiple kernel learning, support vector machines, source selection

## Abstract

Advancement on computer and sensing technologies has generated exponential growth in the data available for the development of systems that support decision-making in fields such as health, entertainment, manufacturing, among others. This fact has made that the fusion of data from multiple and heterogeneous sources became one of the most promising research fields in machine learning. However, in real-world applications, to reduce the number of sources while maintaining optimal system performance is an important task due to the availability of data and implementation costs related to processing, implementation, and development times. In this work, a novel method for the objective selection of relevant information sources in a multimodality system is proposed. This approach takes advantage of the ability of multiple kernel learning (MKL) and the support vector machines (SVM) classifier to perform an optimal fusion of data by assigning weights according to their discriminative value in the classification task; when a kernel is designed for representing each data source, these weights can be used as a measure of their relevance. Moreover, three algorithms for tuning the Gaussian kernel bandwidth in the classifier prediction stage are introduced to reduce the computational cost of searching for an optimal solution; these algorithms are an adaptation of a common technique in unsupervised learning named local scaling. Two real application tasks were used to evaluate the proposed method: the selection of electrodes for a classification task in Brain–Computer Interface (BCI) systems and the selection of relevant Magnetic Resonance Imaging (MRI) sequences for detection of breast cancer. The obtained results show that the proposed method allows the selection of a small number of information sources.

## 1. Introduction

In machine learning, multimodality refers to the simultaneous use of different information sources to solve a specific problem [1]. It is applied to improve some aspects of algorithms, such as the feature generation process or separation between classes, referring specifically to the machine learning area. The use of multimodal sources offers some advantages because it provides additional information about the problem being solved [2]. However, having multiple information sources can also become a problem as the implementation cost can be substantially increased due to the procedural and financial parts of the solution. Currently, different research fields conduct studies that involve the use of multimodal information sources to improve the performance of their works [3,4,5]; however, at the same time, this trend creates the need for studies into the control and optimization of the use of these sources so that the tasks to be carried out are much more efficient in terms of cost, development, and processing times [6,7,8].

Taking into account that in recent times machine learning algorithms have proven to be extremely useful for processing large amounts of data, different studies have been conducted to automatically reduce the amount of data. The most similar approach to the automatic selection of relevant information sources is feature selection through machine learning methods [9]. Such an approach is implemented mainly because an information source can be considered a group of features that share the same nature. The selection of relevant features has been widely addressed in the machine learning field, and it is possible to adapt classical feature selection methods to simultaneously select or delete groups of features, which is known as group feature selection [10,11]. The studies that apply group feature selection usually identify and distinguish the information sources in a specific research area, such as electrodes in a Brain–Computer Interface (BCI) system [12] or different frequency bands in multispectral and hyperspectral imaging [13].

The most basic group feature selection methods use a defined classification threshold; they take a reference value that must be reached to decide whether or not eliminating a feature group [11,14]. Other methods use more advanced procedures to determine the relevance of a set of features based on a penalty imposed during the learning stage of the algorithm [15]; aiming the self method eliminates or reduces the effect of the least relevant feature groups by decreasing the values of the weights assigned to them [14,16]. There is another pair of group feature selection methods known as backward elimination and forward addition [17]; they are usually presented together and consist of removing or adding features to the classification task, and analyzing the performance curve generated during training, seeking to identify the points where the performance of the algorithm is maximum.

Although there is a wide variety of strategies that can be implemented to select feature groups, there is a common challenge all these methods share: the fact that the implementation of algorithms based on single feature selection strategies does not guarantee that the whole information source can be seen as a complete and independent block. That is, these methods usually retain an individual notion of the features to determine an apparent relevance of the information source, thus losing the overall properties of the source and causing breaks between some important relationships between the features that compose the source.

One method that has grown in importance in recent years in the machine learning field, regarding the use of multiple information sources, is Multiple Kernel Learning (MKL) [18,19]. MKL allows the implementation of a similarity measure (kernel) associated with each information source in an independent way before each of those sources is integrated into the learning task [20,21], thus taking advantage of the information coming from each source. Besides, it enables users to obtain easy-to-interpret results in relation to the analysis of each information source [22].

MKL has brought a lot of advantages for different tasks in which it has been implemented, especially when used with the Support Vector Machine (SVM) method [23] as well as with other types of machine learning algorithms [24]. In addition, MKL is well documented in the state of the art and has proven to be useful for the identification and selection of relevant information sources [25,26]. This is because it allows the user to have a similarity measure of data for each information source without losing the possible internal relationships of the features that compose the source, thus enabling objective studies into relevance analyses that are very easy to interpret.

In the state of the art, the use of MKL has been reported when weights are assigned to each kernel associated with the information sources [27], which is an important feature of this methodology since these weights can be used to determine the relevance that each source represents for the implemented solution [28,29,30]. It has been effectively demonstrated that the selection of relevant information sources by penalizing kernel weights is very useful and provides information that is easy to interpret [31], in addition to being a method that can be taken to different research areas.

This work proposes a novel method to objectively select the most relevant information sources in a classification task; this method uses the local scaling technique to tune the parameters of Gaussian kernels associated with each information source by using the MKL. Instead of computing a unique kernel bandwidth for all data, the local scaling technique computes a kernel bandwidth for each sample; thus, it exploits local statistics of sample neighborhood, capturing structure in data [32]. This technique has been used in combination with MKL to perform adaptive unsupervised clustering [33]. In this paper, three different algorithms were also proposed to adapt the local scaling technique to be used during the prediction stage in a supervised classification task, allowing to reduce the computational complexity of the tuning process of kernel parameters considerably. The proposed method is evaluated over two real application tasks: the selection of electrodes for a classification task in Brain–Computer Interface (BCI) systems and the selection of relevant Magnetic Resonance Imaging (MRI) sequences for detection of breast cancer. The obtained results show that the proposed method is stable regarding the sources which are selected as relevant when any of the three proposed algorithms are applied.

## 2. Methodology

This paper proposes a novel method to address the problem of identifying and selecting relevant available information sources by solving a binary classification task in an objective way. The proposed method is based on the use of techniques that have been well studied in the machine learning area, but without the joint implementation reported in the literature. This section contains a detailed explanation of the techniques involved in the proposed method, beginning with the theoretical framework of each technique and finishing with the pseudocode of the algorithms designed to apply such a method.

### 2.1. Support Vector Machines (SVMs)

As mentioned before, the proposed method is only applicable to binary classification problems (for now). This restriction is due to the use of an SVM classifier as base learner in the machine learning task. The SVM is a well-known classifier designed adopting the structural risk minimization theory in order to produce a successful generalization of the prediction using unknown data [34]. The SVM classifier finds the boundary line that better discriminates the training samples contained in a database represented by ${\left\{\left(x_{i},y_{i}\right)\right\}}_{i=1}^{N}$. As a result of this property, SVMs are known as large margin classifiers [35]. Each $x_{i}$ in the database is an input vector of dimension *D*, and y∈{−1,+1} is the label vector which has *N* elements.

The classification function f(x)=〈w,x〉+b defines the distance to the hyperplane which can be seen as a membership degree assigned by the SVM to a test sample, where *w* is the weight associated with each sample $x_{i}$, *b* is the hyperplane bias term, and the 〈·,·〉 operator refers to the dot product between vectors. The primal optimization problem of the SVM is presented in Equation (1), and *w* and *b* are computed when this problem is solved.

(1) $$\begin{array}{cc} \; & w^{*}=\min_{w}\;\frac{1}{2}{∥w∥}_{2}^{2}+C\sum_{i=1}^{N}\xi_{i}\\ \; & s.t.\;y_{i}\left(\langle w,x\rangle +b\right)\geq 1-\xi_{i} \end{array}$$

where *C* is a regularization parameter defined by the user, and ξ is the vector of slack variables.

Lagrange Multipliers are used to solve the quadratic optimization problem in Equation (1) [34], changing the classification function to $f\left(x\right)=\sum_{i=1}^{N}\alpha_{i}y_{i}K\left(x_{i},x\right)+b$ and creating the final objective function presented in Equation (2), namely, the dual function of the SVM.

(2) $$\begin{array}{cc} \; & \max_{0\leq \alpha \leq C}\;\sum_{i=1}^{N}\alpha_{i}-\frac{1}{2}\sum_{i=1}^{N}\sum_{j=1}^{N}\alpha_{i}\alpha_{j}y_{i}y_{j}K(x_{i},x_{j})\\ \; & s.t.\;\sum_{i=1}^{N}\alpha_{i}y_{i}=0 \end{array}$$

where α is the dual variables vector originated by applying Lagrange Multipliers. The term $K(x_{i},x_{j})$ in Equation (2) is known as the kernel function; it also appears in the classification function. It is essential for the performance of the SVM since it generalizes the SVM classifier to solve non-linear problems by allowing the calculation of non-linear decision boundaries. A kernel represents a similarity measure (dot product) between two samples and is commonly expressed as $K(x_{i},x_{j})$, where $K:\;R^{D}\times \;R^{D}⟶R$. Such dot products are made implicitly in a high-dimensional Hilbert Space by using the kernel trick, without the explicit knowledge or use of a mapping function to a high-dimensional space [36].

There are different types of representations of a kernel function, which to be valid and represent dot products on Hilbert spaces must fulfill Mercer’s condition [36]. The simplest one is the dot product ($\langle x_{i},x_{j}\rangle$), namely, linear kernel, and one of the most commonly used is the Gaussian kernel, also known as radial basis function kernel, which is defined by $K\left(x_{i},x_{j}\right)=\exp\left(\frac{-{∥x_{i}-x_{j}∥}_{2}^{2}}{\sigma^{2}}\right)$, where σ is the kernel bandwidth and must be a positive real number.

The algorithm most commonly used to handle the dual function of the SVM is the Sequential Minimal Optimization (SMO) algorithm [37]. There are a lot of programming libraries designed to successfully apply the SMO algorithm, e.g., the LIBSVM library is one the most widely implemented in the literature [38] because it has a cutting-edge repository that can be used over different programming languages and is well supported by the scientific community.

### 2.2. Kernel Bandwidth Tuning with Local Scaling

A recurrent challenge found in the literature is the correct tuning of the σ parameter when a Gaussian kernel is used in the SVM [39,40,41]. This problem has been addressed with different strategies, mainly metaheuristic optimization techniques that consume significant time and computational resources, which is why it remains an open issue in the machine learning area.

Zelnik-Manor and Perona [32] proposed a clever and intuitive method for tuning the σ parameter without using metaheuristic methods. Furthermore, their strategy allowed them to define a different $\sigma_{i}$ value related to each sample in the database instead of a global σ; this strategy is known as local scaling. To compute the local scaling parameter for each $x_{i}$ in the database, it is necessary to analyze the local statistics of its neighborhood because the value of $\sigma_{i}$ is defined by Equation (3).

(3) $$\sigma_{i}=d\left(x_{i},x_{K}\right)$$

where $x_{K}$ is the K-th nearest neighbor of sample $x_{i}$ and d(·,·) is some distance function used to evaluate the local statistics of the data. This work uses the Euclidean distance following the proposal of Zelnik-Manor and Perona [32]. The *K* value determines the neighborhood size and relies on the scale or density of the samples space, i.e., a larger *K* value represents a larger similarity among samples, while a small *K* value focuses on local similarities. Therefore, a new parameter should be tuned when local scaling is used. This parameter is the correct neighbor *K* to compute the distance that defines each $\sigma_{i}$ value.

If any optimization algorithm is used for tuning the σ value in the general radial basis function kernel, it should deal with an infinite searching space of real values. Otherwise, when the local scaling technique is used, even if *K* remains as a free parameter, the optimization problem is reduced to a limited space of integer values considering that K∈Z:1⩽K<N. Besides, the neighborhood of $x_{i}$ will be defined for a small number of samples because using a large *K* value would cause the loss of the local scaling property. When the local scaling strategy is applied, the Gaussian kernel is computed using Equation (4).

(4) $$K\left(x_{i},x_{j}\right)=\exp\left(\frac{-{∥x_{i}-x_{j}∥}_{2}^{2}}{\sigma_{i}\sigma_{j}}\right)$$

### 2.3. Multiple Kernel Learning (MKL)

In recent years, MKL has attracted interest in different research areas, especially because it allows users to take advantage of multiple information sources to solve machine learning problems, even when each information source has a different nature [25,42]. The MKL methodology establishes that multiple linear or non-linear combinations of kernels can be used instead of one single kernel. One of the most commonly used MKL functions is a weighted sum of kernels as shown in Equation (5). This function enables the use of the individual information provided by each data source, in addition to keeping the internal relationships between the features that compose the whole source.

(5) $$K_{\eta }\left(x_{i},x_{j}\right)=\sum_{m=1}^{P}\eta_{m}K_{m}\left(x_{i}^{m},x_{j}^{m}\right)$$

where *P* is the number of information sources that compose the database and $\eta_{m}$ represents the weight assigned to each kernel function $K_{m}$.

An important detail of the MKL function presented in Equation (5) is the possibility of applying a penalization to the weights η in order to identify the most relevant information sources, i.e., the possibility of selecting the sources that provide the most useful information to solve the classification task that is being studied [43]. One of the types of penalization most commonly applied to the η weights is the $\ell_{1}$-norm penalization. The latter provides a straightforward interpretation of results because it fulfills the characteristic of being a sparse penalization type, making some weights equal zero and thus eliminating the least useful information sources [44]. The $\ell_{1}$-norm penalization satisfies the condition η∈Δ, where Δ is the domain of η and is defined by Equation (6), which corresponds to a convex sum, namely, the simplex condition.

(6) $$\Delta =\left\{\eta \in R_{+}^{P}:\sum_{m=1}^{P}\eta_{m}=1,\;\eta_{m}\geq 0\right\}$$

Equation (6) clearly defines how the η weights can be interpreted as relevance measures because each one of them is associated with an individual kernel, which, in turn, is associated with an independent information source. Additionally, all the information sources could be different in terms of nature and number of features without affecting the implementation of the strategy. Kloft et al. [45] and Xu et al. [42] conducted two different studies to find the correct way to train the η weights using a generalized penalization of the $\ell_{p}$-norms with p≥1. Although the analysis in the two studies was performed in a different way, the result was the same. They found an iterative optimization strategy that solves an SVM and updates the kernel weights with Equation (7) until a tolerance measure is satisfied.

(7) $$\eta_{m}=\frac{{∥w_{m}∥}_{2}^{\frac{2}{p+1}}}{{\left(\sum_{h=1}^{P}{∥w_{h}∥}_{2}^{\frac{2p}{p+1}}\right)}^{\frac{1}{p}}}$$

where ${∥w_{m}∥}_{2}^{2}=\eta_{m}^{2}\sum_{i=1}^{N}\sum_{j=1}^{N}\alpha_{i}\alpha_{j}y_{i}y_{j}K_{m}\left(x_{i}^{m},x_{j}^{m}\right)$, which is computed from the dual function of the SVM.

### 2.4. Description of the Proposed Method

**Figure 1:**
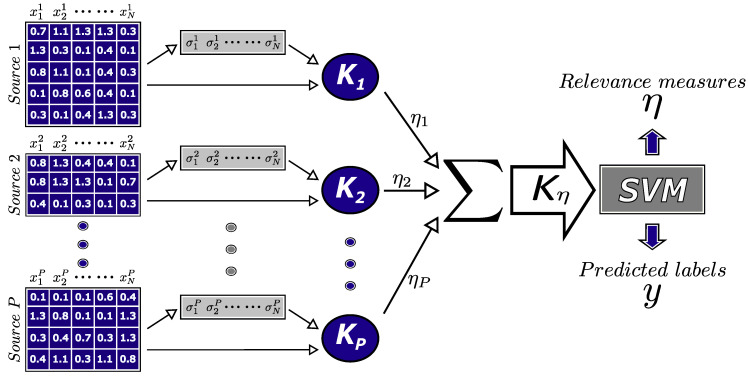
Flowchart of the proposed method.

The proposed method uses MKL to select relevant sources from multimodal data, i.e., the multiple sources available for the classification task. One matrix represents each information source with *N* columns corresponding to the samples and an independent number of rows corresponding to the features. The local scaling technique is used to compute the $\sigma_{i}$ values representing the local statistics of the data, what is necessary to compute the *P* kernels associated with the information sources. Then, the global kernel $K_{\eta }$ obtained after applying the MKL function based on the weighted sum of kernels is fed into an SVM classifier to complete a training process and solve the task. Thus, obtaining a trained predictive model with the definitive η values associated with the relevance measures of the information sources. Figure 1 shows a rough representation of the proposed method as a flowchart.

Because the local scaling strategy was proposed to be applied over unsupervised learning tasks [32], there is no defined process to apply this technique in a supervised learning approach, where labeled data is used to create a predictive model and avoid using all the training information at the prediction stage. Explicitly using the SVM classifier, it is necessary to define a way to compute or estimate each σ value associated with the test samples, contemplating that an SVM only requires the information of some samples (support vectors) to classify unknown data, whereby there is missing information about the original local statistics in the prediction stage. This work proposes three different algorithms to expand local scaling applications to supervised learning tasks; these algorithms are described below.

#### 2.4.1. Prediction without Data Reduction

The first idea that comes to mind in order to avoid this problem is to compute the distance between the test and training samples and assign the σ values to the test samples using the distance of the K-th neighbor. A clear disadvantage of this solution is that all the training samples will be necessary in the prediction stage in order to correctly compute the σ values of the test samples. Algorithm 1 describes how this solution can be applied.
**Algorithm 1:** Algorithm without data reduction
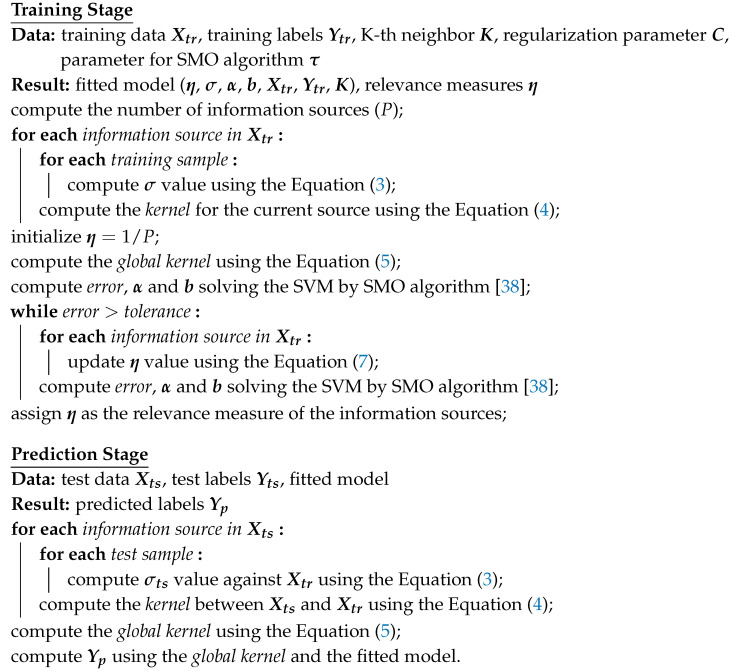


#### 2.4.2. Prediction with Support Vectors Only

Although the first algorithm represents the most accurate approach to compute the σ values of the test samples, it wastes one of the most important characteristics of the SVM: its ability to implement only a few training samples, known as support vectors, in the prediction stage. The second algorithm was designed with the aim of avoiding this issue, using only the σ values of the support vectors. In this algorithm, the distance between the test samples and support vectors is computed, assigning to each test sample the corresponding σ of its nearest support vector. Algorithm 2 describes how this strategy can be applied.
**Algorithm 2:** Algorithm with data reduction by support vectors only
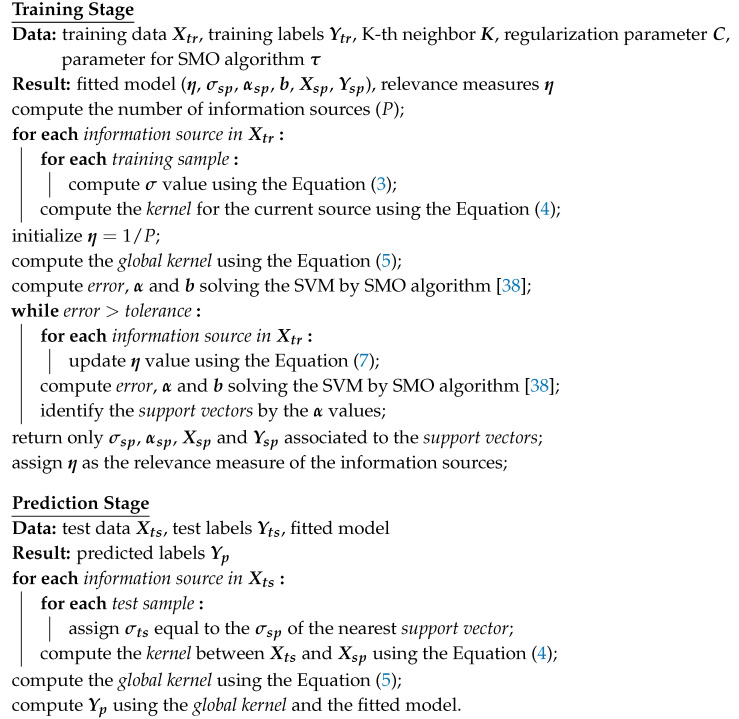


#### 2.4.3. Prediction with Mean σ Values of Support Vectors

The second algorithm presents the best solution with respect to data processing and memory consumption, but it appears to ignore information that belongs to the training samples that were not selected as support vectors and could be important to determine the σ values of the test data. The third algorithm was designed to avoid this issue. In this algorithm, a new step is added to the training stage. It consists in identifying all the training samples that had not been selected as support vectors, assigning to each one of them the nearest support vector, and, finally, computing, for each support vector, a new σ value composed of the mean of the σ values belonging to its nearest training samples. In the prediction stage, the distance between the test samples and support vectors is computed, assigning to each test sample the σ mean associated with its nearest support vector. This process is described in Algorithm 3.
**Algorithm 3:** Data reduction by the mean of σ values related to the samples nearest to the support vectors
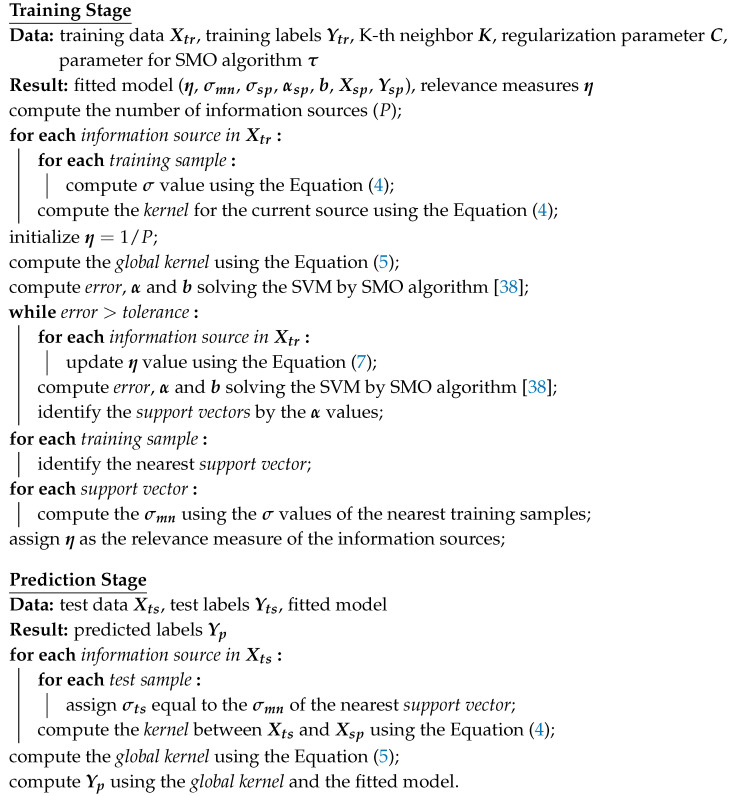


## 3. Experimental Setup

The proposed method can be applied to any well-structured classification task with a multimodal designation. For this reason, we decided to test it in two different tasks and thus demonstrate its usefulness. The first application scenario was a well-known state-of-the-art problem: the selection of relevant electroencephalography (EEG) channels in a Brain–Computer Interface (BCI) classification task. The second scenario was a problem that has not been addressed by the machine learning field: the objective selection of relevant information sources, more specifically, the selection of relevant Magnetic Resonance Imaging (MRI) sequences for breast cancer detection. This section describes the configuration of the datasets and the structure of all the experiments conducted here to evaluate the proposed method.

### 3.1. BCI Dataset

This dataset was published for the BCI competition IV dataset 2a, held in 2008 by the Graz University of Technology in Austria [46]. The dataset is composed of EEG signals taken from 9 healthy subjects. The EEG signals can be categorized into 4 classes of motor imagery: left hand, right hand, feet, and tongue. Each subject completed two sessions on different days; each session consisted of 6 runs separated by short time lapses. A total of 48 trials were recorded in each run (12 per class), thus conducting 288 trials per session. In this study, only the motor imagery of the left and right hands was used since the proposed method is restricted to binary classification problems only. Besides, only the trials of one session were provided with their corresponding labels. Finally, 72 trials were carried out for imaging the movement of the left hand and 72 for the right hand of each subject. In order for the method (which is based on an SVM) to carry out an adequate classification task, the left hand signals were assigned to class 1 and the right hand signals were assigned to class −1.

**Figure 2:**
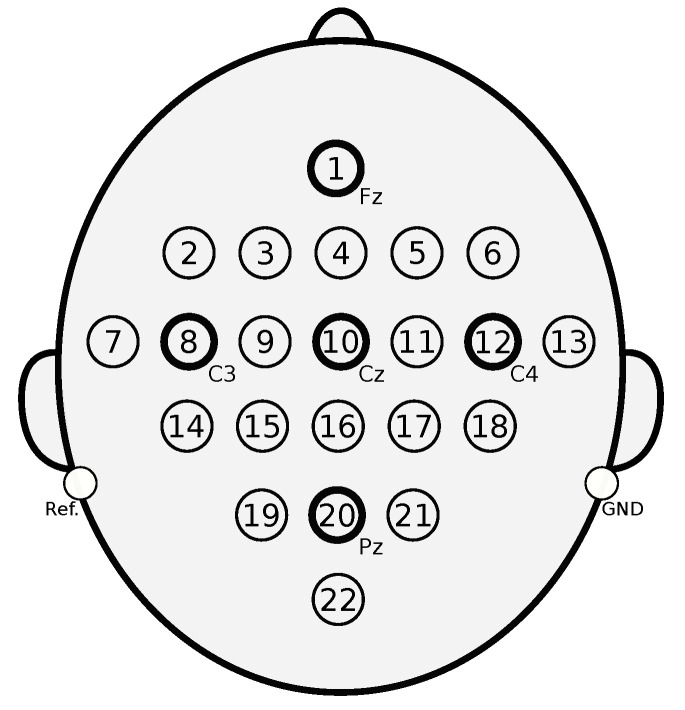
Distribution of electrodes for EEG signals acquisition.

The signals were acquired with a sampling frequency of 250 Hz using 22 electrodes with an inter-electrode distance of 3.5 cm based on the international 10–20 system (see Figure 2). In this case, each electrode was an independent information source that provided descriptive data to be used in the problem solution. Hence, this dataset was composed of 22 information sources in relation to the notation presented in this document. In addition to the 22 electrodes, three electrooculography (EOG) channels were added to the setup during data acquisition. The data provided by these EOG channels were not used as information sources for the classification task; they were only used for applying a preprocessing step to remove artifacts from the EEG signals. Besides, two filters were applied to all the signals (including EOG) as another preprocessing step: (1) a bandpass between 0.5 Hz and 100 Hz and (2) a 50 Hz notch filter.

A feature generation process was applied to the preprocessed signals. Such process allowed us to extract descriptive measures and information to represent the signals in a compressed way. The mother wavelet transformation Daubechies of order 2 (db2) has proven to be efficient in describing EEG signals for classification tasks [47]; thus, it was selected to generate the features of the BCI dataset in this study. Different decomposition levels were considered, taking into account that this dataset is composed of motor imagery signals. As a bandpass filter between 0.5 Hz and 100 Hz was applied to the signals, the detail coefficients of the third (D3) and fourth (D4) decomposition levels are associated with the mu (6.25–12.50 Hz) and beta (12.50–25.00 Hz) rhythms, respectively. These rhythms are commonly used to process motor imagery EEG signals in the state of the art [48,49,50]; hence, they were computed in this dataset. Additionally, the frequency ranges 0.5–3.13 Hz and 3.13–6.25 Hz are also highlighted in the literature. They are associated with approximation (A5) and detail (D5) coefficients of the fifth decomposition level, respectively [50]. Thus, they were also used in this study.

After computing the wavelet coefficients D3, D4, D5, and A5, six statistical measures of each coefficient and the original signal were calculated: mean, median, mode, variance, kurtosis, and standard deviation [51]. Additionally, the energy of each coefficient and the original signal was also computed. As a result, we obtained a total of 35 features per information source.

### 3.2. Breast Cancer Dataset

**Figure 3:**
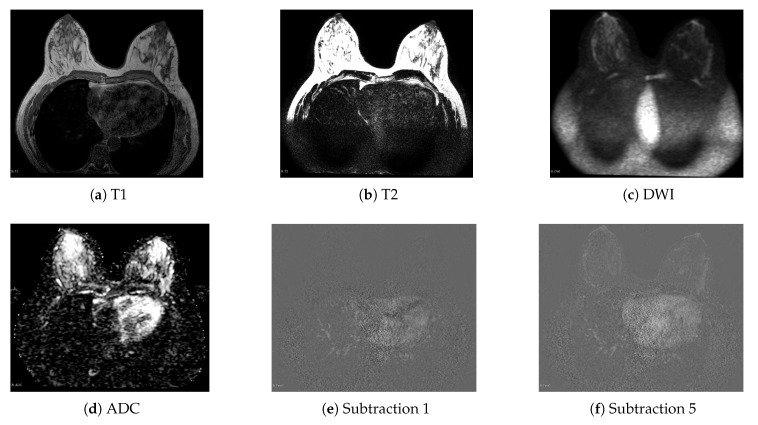
Visualization of the MRI sequences in the breast cancer dataset.

The MRI breast cancer dataset was developed by Instituto Tecnológico Metropolitano (ITM) in collaboration with Instituto de Alta Tecnología Médica (IATM) in Medellín, Colombia. It is composed of 87 studies, and each study corresponds to one subject and presents at least one Region of Interest (ROI). In total, 146 ROIs were extracted from the entire dataset. Each study in the dataset is represented by nine information sources, and each source corresponds to an MRI sequence. The sequences included in the dataset are the relaxations T1 and T2, Diffusion-Weighted Imaging (DWI), Apparent Diffusion Coefficient (ADC), and subtractions 1 to 5 obtained from Dynamic Contrast Enhanced (DCE) sequences [52]. Each MRI sequence highlights different three-dimensional information from the tissue under analysis so that it can be ensured that each sequence corresponds to an independent information source. Figure 3 shows an example of the visualization of these sequences.

The complete dataset was evaluated by two expert radiologists from IATM, who classified each finding in the sequences using the Breast Imaging Reporting and Data System (BI-RADS) [53]. This system enabled them to assign a probability level of a finding to be cancer, where 5 is the highest level: highly suggestive of malignancy. In order to adapt the dataset to the classification method proposed in this paper, the ROIs were separated into two classes: class −1 for all the ROIs with a BI-RADS less than 3 and class 1 for the ROIs with a BI-RADS equal to or greater than 3. A total of 61 ROIs were associated with class −1 and 85 with class 1 applying this configuration.

Two different set of features were implemented to describe the information of the findings in each sequence, i.e., perceptual and radiomic features. Perceptual features refer to the properties of an image that can be captured by human perception [54], while radiomic features represent a quantitative approach to the analysis of medical images aimed at explaining the morphological and functional properties of a lesion [55].

A total of 10 perceptual features and 86 radiomic features were computed for each image sequence. Perceptual features were computed as the first five statistical moments from both the salient maps generated by the Graph-Based Visual Saliency method [56,57] and the original images; while radiomic features were extracted using the Pyradiomics toolbox of Python [58]. As described below, the experimental stage was performed by considering both each set of features as an independent information source and the combination of the two sets of features as one unique information source. Thus, a total of 18 information sources per instance were obtained, i.e., nine sources that correspond to the set of perceptual features for each sequence (P_T1, P_T2, P_ADC, P_DWI, P_SUB1, P_SUB2, P_SUB3, P_SUB4, and nine other sources that correspond to each set of radiomic features (R_T1, R_T2, R_ADC, R_DWI, R_SUB1, R_SUB2, R_SUB3, R_SUB4 and R_SUB5).

The main objective of integrating perceptual and radiomic features in this study is to obtain relevant results for both machines and radiologists.

### 3.3. Test Settings

The tests conducted here to evaluate the performance of the proposed method take into account different variations that it may present. They are mainly associated with the three algorithms proposed to adapt the local scaling method to the supervised learning task, the types of features generated from the MRI sequences, the number of subjects in the BCI dataset, and the two main types of penalties that can be applied to the η weights associated with the kernels (which correspond to the $\ell_{1}$-norm and the $\ell_{2}$-norm). Therefore, it is necessary to explain how the tests were configured for each dataset.

#### 3.3.1. BCI Dataset Configuration

The tests performed using the BCI dataset were divided into two large groups determined by the penalty types ($\ell_{1}$-norm and $\ell_{2}$-norm). Then, each of these groups was divided into three subgroups determined by the three algorithms proposed here to adapt the local scaling method. Finally, a test was conducted on each subgroup using each one of the nine subjects that compose the dataset. This resulted in a total of 54 different tests taken into account to evaluate the performance of the proposed method with the BCI dataset.

#### 3.3.2. MRI Dataset Configuration

In the same way, as with the BCI dataset, the tests with the MRI dataset were divided into two large groups determined by the penalty types ($\ell_{1}$-norm and $\ell_{2}$-norm). Then, each test group was divided into three subgroups determined by the available feature types. The first subgroup included only perceptual features; the second, only radiomic features; and the third, both feature types. Finally, a test was applied to each subgroup using each one of the three proposed algorithms. This resulted in a total of 18 different tests taken into account to evaluate the performance of the proposed method with the MRI dataset.

#### 3.3.3. General Configuration of the Tests

Although the purpose of the proposed method is the objective selection of relevant information sources, it is necessary to evaluate the effectiveness of the method based on its performance in terms of the classification task. Therefore, we decided to implement 4 well-known performance measures for classification tasks [59]: accuracy, sensitivity, specificity, and geometric mean (Geo-Mean). They were computed using Equations (8) to (11), respectively.

(8) $$\mathrm{Accuracy}=\frac{\mathrm{Correctly}\;\mathrm{predicted}\;\mathrm{samples}}{\mathrm{Total}\;\mathrm{number}\;\mathrm{of}\;\mathrm{samples}}$$

(9) $$\mathrm{Sensitivity}=\frac{\mathrm{True}\;\mathrm{positives}}{\mathrm{True}\;\mathrm{positives}\;+\;\mathrm{False}\;\mathrm{negatives}}$$

(10) $$\mathrm{Specificity}=\frac{\mathrm{True}\;\mathrm{negatives}}{\mathrm{True}\;\mathrm{negatives}\;+\;\mathrm{False}\;\mathrm{positives}}$$

(11) $$\mathrm{Geo}-\mathrm{Mean}=\sqrt{\mathrm{Sensitivity}\;\cdot \;\mathrm{Specificity}}$$

Each dataset was divided maintaining a 20% of the samples outside the training and validation stage to use them as test samples, while the remaining 80% was used to train the model. For the training and validation process, the cross-validation method K-Fold was implemented with a total of 10 folds in order to be able to train the method in such a way that it could correctly generalize the predictions about test data.

Although the method proposed by Zelnik-Manor and Perona [32] set the *K* value to 7 because it produced good general results, in this work, *K* was defined as a parameter to be optimized, in addition to the regularization parameter *C*, and the parameter for the SMO algorithm τ. The Particle Swarm Optimization (PSO) algorithm was implemented to find the optimal values of these parameters. The PSO is one of the most commonly used optimization algorithms in the state of the art [60,61,62]. It uses cooperative and stochastic methods to find the optimal parameters of the function to be optimized; in this case, the performance of the classifier. In all the tests, we used 40 search particles and individual and social learning coefficients equal to 1.1931 [63], and the inertia value was dynamically established between 0.1 and 1.1 with a maximum value of 60 iterations. The cost function used for the optimization process was the geometric mean because it was the performance measure that showed the best stability and quick convergence regarding the optimal values that are found.

Once the optimization process was completed using the PSO and cross-validation in each test, the optimal parameters found were used to train a global model using the total training data. This model was finally employed to make the prediction on the test data that had been initially separated, thus obtaining the trained values of the η weights that determine the relevance of the information sources and the predictions on the test data that would allow us to measure the performance of the method as a classifier.

Furthermore, Equation (12) was implemented in order to measure the effectiveness of the method in terms of the reduction rate of the number of information sources, taking into account that the ideal situation is to obtain a high reduction rate because it means the use of a minimal number of sources to solve the classification task.

(12) $$\mathrm{Reduction}\;\mathrm{rate}=1-\frac{\mathrm{Number}\;\mathrm{of}\;\mathrm{sources}\;\mathrm{selected}}{\mathrm{Total}\;\mathrm{number}\;\mathrm{of}\;\mathrm{sources}}$$

## 4. Results and Discussion

This section reports the results obtained with the BCI and the MRI datasets. Such results are analyzed in detail and discussed taking into account some relevant studies in the literature that present reference points to assess the performance of the proposed method.

### 4.1. Results Obtained with the BCI Dataset

A total of 54 tests were conducted with this dataset, as described in the previous section. After completing the training stage and obtaining the optimal values for the free parameters, a prediction process was performed with the test data while a sequential reduction as applied to the number of information sources. This was possible because the global model obtained after the training stage contained the η values of each information source (each electrode), and these values are directly associated with the relevance measure obtained for each source. As a result, the most relevant information sources can be identified and sequentially eliminated from least to most relevant when the η values are sorted from highest to lowest.

**Figure 4:**
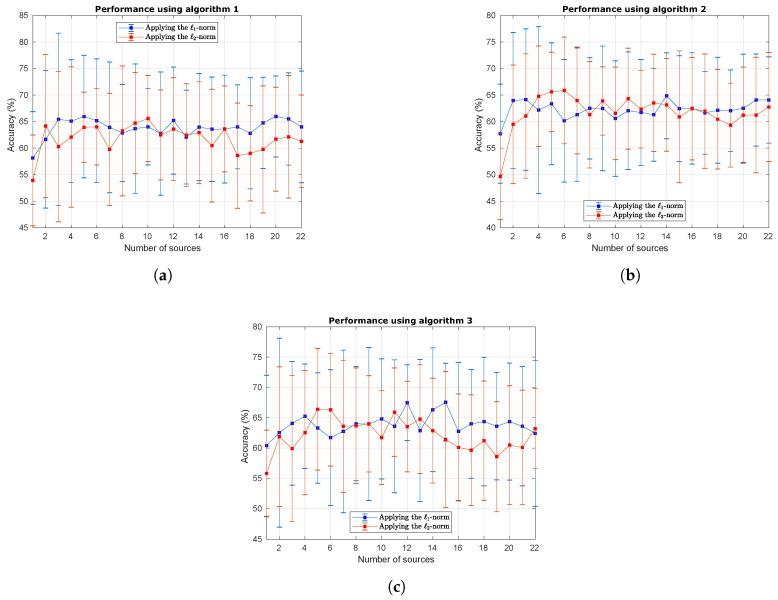
Performance curves obtained with the BCI test dataset when the number of electrodes was reduced. (**a**) Performance curves of Algorithm 1; (**b**) Performance curves of Algorithm 2; (**c**) Performance curves of Algorithm 3.

Figure 4 shows the curves obtained with the process of reduction of information sources. Taking into account that there is a lot of information, the results were condensed in three different figures that represent the application of the three proposed algorithms. Such figures show the average value and standard deviation of the accuracy obtained for the nine subjects, separated by the application of the $\ell_{1}$-norm and $\ell_{2}$-norm.

Figure 4a shows the performance curves of Algorithm 1. In it, the highest mean performance with the lowest number of sources is obtained using the $\ell_{1}$-norm; nevertheless, the $\ell_{2}$-norm shows a very similar behavior, reaching a high performance when only 2 information sources are used. Figure 4b shows the performance curves of Algorithm 2. In this case, the $\ell_{2}$-norm achieves the highest performance with the lowest number of sources, more specifically, between 4 and 6 sources. Finally, Figure 4c shows the performance curves of Algorithm 3. Again, both the $\ell_{1}$-norm and the $\ell_{2}$-norm exhibit similar behavior, although the $\ell_{2}$-norm slightly outperforms the $\ell_{1}$-norm when only 5 information sources are used. Based on these results, we decided to include only the detailed performance specifications of the method using the $\ell_{2}$-norm because the performance of both penalization types was very similar, and including all the detailed performance results could seem redundant.

Table 1 details the classification performance results obtained with the BCI dataset when Algorithm 1 was applied with the $\ell_{2}$-norm. Said table compares the results obtained when the 22 information sources were used and the best result obtained per subject with the lowest number of sources that could be used. These sources are sorted by relevance from high to low. Evidently, the performance measures always improve when the number of sources is reduced. It is also important to highlight that each subject requires a different number of information sources and that the selected electrodes are different for each subject (with some coincidences in several cases). Table 1 also shows the time required by the computer to train the global model and make predictions with the test data. All these tests were conducted on a work station with an Intel processor of 16 cores at 3.00 GHz and 16 GB of RAM. The last column in the table shows the number of samples required by the algorithm to compute the prediction with the test data. In this case, all the training samples were required by the algorithm because that is precisely the main feature of Algorithm 1 and also its worst disadvantage. The last row shows the average computed using all the information obtained from all the subjects with its corresponding standard deviation. The relevant electrodes were selected by analyzing which of them were the most voted among all the subjects.

Table 2 details the results obtained with the BCI dataset when Algorithm 2 was used with the $\ell_{2}$-norm. These results are very similar to those obtained in Table 1. The most important aspect to highlight in this table is the fact that all the most relevant electrodes selected in the average results were also selected by Algorithm 1, which means high stability in the method among the algorithms. Furthermore, the results show again that the proposed method can improve the performance (measures) of the classification task and, at the same time, produce a reduction in the required number of samples (nearly 72%) because this algorithm only uses the support vectors to make predictions.

Table 3 presents the results of the method applied to the BCI dataset when Algorithm 3 and the $\ell_{2}$-norm are used. The electrodes selected as relevant are the same as in Table 1 and Table 2, thus confirming, once again, the stability of the method in terms of the selection of relevant information sources even when the type of algorithm is different. Table 3 also shows that Algorithm 3 reduces the number of samples required for the prediction by nearly 74%. We should remember that this algorithm takes the information of the samples that the SVM does not select as support vectors for computing mean sigma values and takes advantage of all the available data.

Taking into account that the stability and reliability of the relevant information sources selected by the method are the most critical factors to analyze, we created Table 4 with the average relevance assigned to each electrode among all the subjects. The accuracy obtained in the classification of each subject was used to weight this relevance, considering that the electrodes selected as relevant by the method are more reliable when the obtained accuracy is higher. In Table 4, it can be seen that the method selected electrodes 8, 13, 14, 18, and 22 as relevant in all the configurations among all the algorithm and penalization types. This table was organized based on the information in Table 1, Table 2 and Table 3, by taking the first eight selected electrodes as relevant. Furthermore, if the penalization type is used to analyze the results presented in Table 4 separately, it can also be seen that electrode 7 was classified as relevant with the $\ell_{1}$-norm; and electrode 1, with the $\ell_{2}$-norm.

### 4.2. Results Obtained with the MRI Dataset

**Figure 5:**
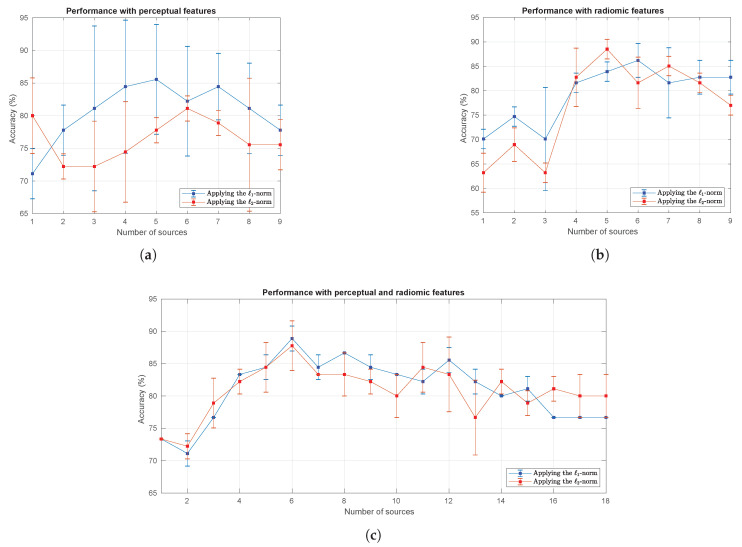
Performance curves obtained with the test dataset when the number of MRI sequences was reduced. (**a**) Performance curves using only the perceptual features; (**b**) Performance curves using only the radiomic features; (**c**) Performance curves using both perceptual and radiomic features.

A total of 18 tests were conducted with the MRI dataset. As in the tests applied to the BCI dataset, a prediction process was performed on the test data while making a sequential reduction in the number of information sources after completing the training stage and obtaining the optimal values for the free parameters. Figure 5 shows the curves obtained with the process of reduction of information sources on the MRI dataset. Three different figures that represent the use of the perceptual features, radiomic features and a combination of both summarize the results. The figures show the average value and standard deviation of the accuracy obtained with the three algorithms, separated by the application of the $\ell_{1}$-norm and $\ell_{2}$-norm.

Figure 5a shows the performance curves obtained using only the perceptual features. In this figure, the $\ell_{1}$-norm clearly outperforms the $\ell_{2}$-norm, reaching the highest mean accuracy with only 5 information sources, which slowly decreases with fewer sources. Figure 5b shows the performance curves obtained using only the radiomic features. In this case, both the $\ell_{1}$-norm and the $\ell_{2}$-norm present a similar behavior, although the $\ell_{2}$-norm slightly outperforms the $\ell_{1}$-norm when 5 information sources are used. Finally, Figure 5c shows the performance curves using perceptual and radiomic features simultaneously. Again, both the $\ell_{1}$-norm and the $\ell_{2}$-norm exhibit a similar performance, and the $\ell_{1}$-norm slightly outperforms its counterpart when only 6 information sources are used. Based on these results, we decided to include only the detailed performance results of the method using the $\ell_{1}$-norm, although the results were remarkably similar to the two penalization types.

Table 5 details the results of the method using only the perceptual features and applying the $\ell_{1}$-norm. This table presents different performance measures obtained when all the information sources and only the most relevant information sources for each algorithm are implemented. In this case, the table specifies the sources selected as relevant to represent the MRI sequences and also indicates the corresponding reduction rate. Additionally, Table 5 shows the time needed by the algorithm to complete the training stage of the global model and the prediction stage with the training and test data. The last column shows the number of training samples that were needed to build the global model (support vectors).

It is evident in Table 5 that identifying which information sources are relevant for the classification task can significantly improve its performance. Algorithm 2 also presents better performance than the other two algorithms, obtaining the best results in all the performance measures when source reduction was applied. Note the coincidence in the information sources selected as relevant by Algorithms 2 and 3, which show a very similar performance that is also better than that of Algorithm 1.

Table 6 details the results obtained using the method with the $\ell_{1}$-norm and only the information sources obtained from the generation of radiomic features on the MRI sequences. In this case, the reduction rate was considerably lower with all the algorithms compared to the previous table, but again the algorithms showed appropriate stability regarding the sources selected as relevant. In addition, Algorithms 2 and 3 present an advantage, especially because they considerably reduce the amount of data that they require for the construction of the final model.

Finally, the results in Table 7 take into account a higher number of information sources because they combine the sources obtained from the generation of perceptual and radiomic features. In this case, Table 7 shows very similar results with the three algorithms, a reduction rate of more than half of the information sources with all the algorithms, and an almost perfect match of the information sources selected as relevant. It is possible to identify highly relevant measures in the MRI sequences represented by subtractions in most tests, with both the perceptual and radiomic features.

### 4.3. Comparison against Feature Selection Methods

The closest approaches to the automatic selection of sources using machine learning are the feature selection techniques developed in said research area [64]. It is possible to associate the selection of information sources with the selection of features, considering that it is reasonable to select individual features hoping to eliminate all the features of one specific information source. Hence, the feature selection technique could eliminate the whole information source.

A new group of tests was performed based on the above and aims to evaluate the contribution of the proposed method to the objective selection of information sources provided for a binary classification task (compared to classical feature selection techniques). These tests consisted of two different experiments: (1) a feature selection process applied to all the information sources in order to automatically eliminate some of these sources if the feature selection process eliminated all the features that composed them; and (2) the application of the proposed method to reduce the number of information sources and the subsequent implementation of the feature selection technique for reducing the number of features that composed the relevant information sources.

The technique used to perform the feature selection process was the Fisher Score [65]. This method is a supervised feature selection technique that seeks to preserve features that have a more uniform distribution in one class and a more dispersed one in the others. The Fisher Score method is represented by Equation (13).

(13) $$FS\left(f_{i}\right)=\frac{\sum_{j=1}^{l}n_{j}{\left(\mu_{i,j}-\mu_{i}\right)}^{2}}{\sum_{j=1}^{l}n_{j}s_{i,j}^{2}}$$

where *l* is the number of classes; $\mu_{i}$, the mean of the feature $f_{i}$; $n_{j}$, the number of samples in the *j*-th class; and $\mu_{i,j}$ and $s_{i,j}$, the mean and the variance of $f_{i}$ in the *j*-th class, respectively [66].

Equation (13) is used to obtain a rank vector ordering the features from the most relevant to the least relevant. Once the features are ordered, they are eliminated one by one starting by the least relevant till identifying the number of features that generate the highest classification performance.

In the first experiment, the whole features extracted from all information sources are concatenated in a unique feature vector before applying the feature selection and source elimination process. Thus, the relevant information sources for the classification task will be those that retain at least one of its features in the set of features that reported the best performance. In the second experiment, the proposed source selection method is applied to find the most relevant information sources. Then, features extracted from these sources are concatenated in a unique feature vector, which is used as input to the feature selection process, in order to select the most discriminative features that improve the classification task. In this case, the relevant sources could also be reduced, by applying the same process described to the first experiment; however, all sources selected initially by the proposed approach were kept in the experiments performed in this work.

Table 8 shows the results obtained with the BCI dataset. The left side of the table groups the results of the first experiment; the right side, those of the second experiment. It can be seen that the feature selection technique improves the performance of the classifier and manages to eliminate some information sources for some subjects by itself in some cases. However, in most cases, it cannot reduce the number of information sources, even with a considerable reduction in the number of features. On the other hand, the right side of the table shows that the proposed method manages to reduce the number of information sources for all the subjects with the three proposed algorithms and, at the same time, maintains high performance in the classification task. These results also indicate that the proposed method can be used to reduce the number of information sources and, after that, a feature selection technique can be applied to reduce the number of features; the objective is to achieve the best results in terms of the highest performance with minimum data quantity.

Table 9 shows the results obtained with the MRI dataset. The results of the first experiment of tests are on the left side; and those of the second experiment, on the right side. It is essential to highlight that the tests achieved the highest performance regarding the classification task when only the feature selection technique was applied, but in this case the task required the use of all the information sources, missing the main objective of the tests. Conversely, the results on the right side of the table show that the proposed algorithm maintained a high performance during all the tests while it eliminated some information sources (more than half in most tests), successfully achieving the proposed objective.

The sources selected as relevant during this group of tests were the same as those presented in the results in Section 4.1 and Section 4.2, which again highlights the stability of the proposed method regarding the objective selection of relevant information sources. Moreover, additional tests were conducted to consider the application of the feature selection technique before reducing the information sources following the structure of tests carried out in some studies in the literature. However, the results of those tests are not reported in this paper because they exhibited the same behavior regarding the information sources selected as relevant and the performance measures they achieved, confirming the stability of the proposed algorithm remains in all the evaluation environments considered here.

## 5. Conclusions

This work proposes an automatic method that implements different types of machine learning algorithms to objectively select the most relevant information sources in a classification task. The proposed method computes a Gaussian kernel for each information source, performing the tuning of the kernel parameters by using the local scaling technique. This technique was adapted through three different algorithms to be used during the prediction stage in the supervised classification task implemented over an SVM. Algorithm 1 uses all the training samples to compute the kernel bandwidth parameter (σ) corresponding to each prediction sample. Algorithm 2 uses only the SVM support vectors to determine the σ, thus reducing the amount of information required by the trained model. In contrast, Algorithm 3 collects the neighborhood information of the support vectors to estimate the σ value of each prediction sample, attempting to keep the information of training samples that are not selected as support vectors while reducing the information required by the trained model.

Two real application tasks were used to evaluate the proposed method: the selection of electrodes for a classification task in Brain–Computer Interface (BCI) systems and the selection of relevant Magnetic Resonance Imaging (MRI) sequences for detection of breast cancer. The obtained results show that the proposed method is stable regarding the sources which are selected as relevant, even when the results are analyzed among the three proposed algorithms. Although the three algorithms presented similar performances and fulfilled the expected function of source selection, it is possible to find a relatively superior performance of Algorithm 2 when using the $\ell_{1}$-norm and likewise, a leading behavior of Algorithm 3 when using the $\ell_{2}$-norm. These two algorithms have the particular advantage of reducing the number of samples required to build the trained model since they only require the information of the samples selected by the SVM as support vectors.

## Figures and Tables

**Table 1:** Results of selection of BCI channels applying the Algorithm 1 and $\ell_{2}$-norm.

Subject	Number of Sources	Performance Measures (%)				Reduction Rate	Relevant Electrodes	Required Time (s)	Support Vectors
		Acc	Geo-M	Sens	Spec				
**1**	22	64.29	63.89	71.43	57.14	0.14	14, 13, 18, 1, 22, 8, 7, 19, 9, 6, 4, 21, 5, 10, 17, 12, 2, 16, 15	0.139	116 (100.0 %)
	19	75.00	74.91	78.57	71.43				
**2**	22	53.33	51.64	40.00	66.67	0.59	13, 1, 12, 6, 20, 7, 3, 8, 17	0.090	116 (100.0 %)
	9	76.67	76.01	66.67	86.67				
**3**	22	60.71	59.76	71.43	50.00	0.91	8, 13	0.016	116 (100.0 %)
	2	89.29	88.64	78.57	100.00				
**4**	22	57.14	56.69	64.29	50.00	0.91	21, 22	0.015	116 (100.0 %)
	2	75.00	72.84	92.86	57.14				
**5**	22	60.00	58.50	73.33	46.67	0.82	3, 22, 1, 13	0.030	116 (100.0 %)
	4	63.33	62.54	73.33	53.33				
**6**	22	63.33	63.25	66.67	60.00	0.41	18, 7, 1, 8, 12, 10, 17, 3, 11, 9, 6, 14, 13	0.089	116 (100.0 %)
	13	70.00	69.92	66.67	73.33				
**7**	22	46.43	46.29	50.00	42.86	0.91	22, 3	0.016	116 (100.0 %)
	2	67.86	67.76	64.29	71.43				
**8**	22	75.00	74.91	78.57	71.43	0.82	18, 14, 6, 13	0.029	116 (100.0 %)
	4	85.71	85.42	92.86	78.57				
**9**	22	71.43	71.43	71.43	71.43	0.27	18, 13, 12, 7, 2, 1, 6, 3, 22, 21, 20, 19, 4, 8, 15, 14	0.115	116 (100.0 %)
	16	78.57	78.25	85.71	71.43				
**Average**	22	61.30 ±8.71	60.71 ±8.98	65.24 ±12.40	57.36 ±10.74	0.64 ±0.30	13, 8, 6, 1, 22, 3, 14, 18	0.060 ±0.048	116 (100.0 %)
	8 ±7	75.71 ±8.23	75.14 ±8.25	77.73 ±11.00	73.70 ±14.09				

**Table 2:** Results of selection of BCI channels applying the Algorithm 2 and $\ell_{2}$-norm.

Subject	Number of Sources	Performance Measures (%)				Reduction Rate	Relevant Electrodes	Required Time (s)	Support Vectors
		Acc	Geo-M	Sens	Spec				
**1**	22	67.86	67.76	71.43	64.29	0.50	14, 13, 18, 1, 8, 22, 19, 21, 9, 10, 4	0.065	83 (71.6 %)
	11	75.00	74.91	78.57	71.43				
**2**	22	66.67	65.32	53.33	80.00	0.36	12, 8, 13, 1, 17, 7, 16, 20, 21, 2, 6, 14, 9, 10	0.096	110 (94.8 %)
	14	66.67	65.32	53.33	80.00				
**3**	22	71.43	71.07	64.29	78.57	0.82	8, 5, 13, 14	0.024	75 (64.7 %)
	4	75.00	74.91	71.43	78.57				
**4**	22	46.43	46.29	50.00	42.86	0.91	22, 21	0.017	72 (62.1 %)
	2	78.57	78.57	78.57	78.57				
**5**	22	56.67	54.16	73.33	40.00	0.77	7, 1, 13, 3, 22	0.030	78 (67.2 %)
	5	66.67	65.32	80.00	53.33				
**6**	22	70.00	69.92	66.67	73.33	0.55	18, 8, 1, 7, 12, 17, 3, 9, 10, 14	0.066	99 (85.3 %)
	10	76.67	76.59	80.00	73.33				
**7**	22	46.43	46.29	50.00	42.86	0.91	22, 3	0.013	83 (71.6 %)
	2	67.86	67.76	64.29	71.43				
**8**	22	67.86	67.76	71.43	64.29	0.82	18, 14, 1, 13	0.028	86 (74.1 %)
	4	82.14	82.07	78.57	85.71				
**9**	22	71.43	71.43	71.43	71.43	0.73	13, 2, 12, 22, 1, 18	0.035	64 (55.2 %)
	6	78.57	78.25	85.71	71.43				
**Average**	22	62.75 ±10.25	62.22 ±10.40	63.55 ±9.77	61.96 ±15.98	0.71 ±0.19	1, 13, 14, 22, 18, 8	0.042 ±0.028	83 ±14 (71.8 % ±12.1 %)
	6 ±4	74.13 ±5.73	73.74 ±6.14	74.50 ±9.99	73.76 ±9.10				

**Table 3:** Results of selection of BCI channels applying the Algorithm 3 and $\ell_{2}$-norm.

Subject	Number of Sources	Performance Measures (%)				Reduction Rate	Relevant Electrodes	Required Time (s)	Support Vectors
		Acc	Geo-M	Sens	Spec				
**1**	22	67.86	67.76	71.43	64.29	0.77	14, 13, 18, 9, 1	0.041	78 (67.2 %)
	5	78.57	78.25	85.71	71.43				
**2**	22	63.33	62.54	53.33	73.33	0.05	7, 1, 13, 6, 8, 9, 12, 2, 3, 14, 18, 17, 20, 10, 5, 21, 19, 15, 16, 11, 22	0.171	105 (90.5 %)
	21	63.33	62.54	53.33	73.33				
**3**	22	64.29	64.29	64.29	64.29	0.91	8, 13	0.017	78 (67.2 %)
	2	82.14	82.07	85.71	78.57				
**4**	22	50.00	50.00	50.00	50.00	0.91	22, 21	0.018	99 (85.3 %)
	2	71.43	71.43	71.43	71.43				
**5**	22	56.67	54.16	73.33	40.00	0.77	7, 1, 13, 3, 22	0.039	78 (67.2 %)
	5	66.67	65.32	80.00	53.33				
**6**	22	66.67	66.33	73.33	60.00	0.50	18, 7, 8, 12, 1, 9, 17, 10, 3, 11, 14	0.090	100 (86.2 %)
	11	73.33	73.33	73.33	73.33				
**7**	22	60.71	60.61	64.29	57.14	0.91	22, 3	0.017	83 (71.6 %)
	2	64.29	64.29	64.29	64.29				
**8**	22	67.86	67.76	64.29	71.43	0.77	18, 13, 14, 9, 1	0.044	83 (71.6 %)
	5	82.14	82.07	85.71	78.57				
**9**	22	71.43	71.43	71.43	71.43	0.73	13, 2, 12, 22, 1, 18	0.045	64 (55.2 %)
	6	71.43	71.43	71.43	71.43				
**Average**	22	63.20 ±6.59	62.76 ±6.91	65.08 ±8.55	61.32 ±11.00	0.70 ±0.28	13, 1, 18, 22, 9, 14, 3	0.054 ±0.049	85 ±13 (73.6 % ±11.5 %)
	7 ±6	72.59 ±7.17	72.30 ±7.39	74.55 ±11.07	70.63 ±7.76				

**Table 4:** Results of selection of BCI channels applying the Algorithms 1 to 3 with $\ell_{1}$-norm and $\ell_{2}$-norm.

Using the $\ell_{1}$-Norm						Using the $\ell_{2}$-Norm					
Algorithm 1		Algorithm 2		Algorithm 3		Algorithm 1		Algorithm 2		Algorithm 3	
Electrode	Relev	Electrode	Relev	Electrode	Relev	Electrode	Relev	Electrode	Relev	Electrode	Relev
**18**	8.95	**14**	10.22	**13**	8.00	**13**	10.08	1	10.48	**13**	10.74
**14**	7.46	**18**	9.23	**14**	7.87	**8**	7.29	**13**	10.45	1	10.52
**13**	7.31	**13**	7.17	**18**	6.97	6	7.22	**14**	8.83	**18**	8.91
**8**	6.09	**22**	7.08	**8**	6.88	1	6.81	**22**	8.63	**22**	8.15
12	6.09	1	6.47	7	6.61	**22**	6.73	**18**	7.35	9	7.19
7	5.98	21	6.36	12	6.61	3	6.67	**8**	6.90	**14**	7.19
**22**	5.92	7	6.27	**22**	5.38	**14**	5.79	12	5.22	3	6.47
10	5.87	**8**	5.77	6	5.32	**18**	5.79	21	5.18	**8**	5.29
21	5.81	6	5.59	1	5.25	7	5.62	9	5.14	12	5.03
11	4.40	12	5.28	19	5.23	12	5.62	10	5.14	7	4.91
17	4.40	9	4.87	10	4.13	21	4.28	3	4.97	10	3.30
6	4.31	2	3.40	17	3.99	17	4.15	7	4.94	11	3.30
3	4.27	15	3.40	11	3.97	20	2.91	2	3.42	17	3.30
2	4.16	19	3.40	15	3.88	2	2.87	17	3.37	2	3.26
1	4.16	11	3.11	3	3.68	4	2.87	4	1.76	21	3.26
5	2.69	20	3.11	2	2.68	15	2.87	5	1.76	5	1.53
9	2.69	4	1.75	5	2.48	19	2.87	19	1.76	6	1.53
19	2.69	3	1.56	21	2.48	9	2.71	6	1.57	15	1.53
20	2.69	10	1.56	9	2.46	10	2.71	16	1.57	16	1.53
15	1.37	16	1.56	16	2.46	5	1.40	20	1.57	19	1.53
16	1.37	17	1.56	20	2.46	16	1.40	11	0.00	20	1.53
4	1.32	5	1.32	4	1.22	11	1.31	15	0.00	4	0.00

**Table 5:** Results of selection of MRI sequences with perceptual features and $\ell_{1}$-norm.

	Performance Measures	Using All Sources (%)	Applying Sources Reduction (%)	Reduction Rate	Relevant Sequences	Required Time (s)	Support Vectors
**Algorithm 1**	Accuracy	73.33	80.00	0.78	P_ADC, P_SUB4	0.013	117 (100.0 %)
	Geo-Mean	65.91	79.59				
	Sensitivity	94.12	82.35				
	Specificity	46.15	76.92				
**Algorithm 2**	Accuracy	80.00	93.33	0.56	P_SUB2, P_SUB4, P_SUB3, P_SUB5	0.021	97 (82.9 %)
	Geo-Mean	76.10	93.21				
	Sensitivity	94.12	94.12				
	Specificity	61.54	92.31				
**Algorithm 3**	Accuracy	80.00	86.67	0.67	P_SUB2, P_SUB5 P_SUB3	0.020	97 (82.9 %)
	Geo-Mean	76.10	85.09				
	Sensitivity	94.12	94.12				
	Specificity	61.54	76.92				

**Table 6:** Results of selection of MRI sequences with radiomic features and $\ell_{1}$-norm.

	Performance Measures	Using All Sources (%)	Applying Sources Reduction (%)	Reduction Rate	Relevant Sequences	Required Time (s)	Support Vectors
**Algorithm 1**	Accuracy	82.76	86.21	0.33	R_T2, R_T1 R_DWI, R_SUB3 R_SUB5, R_SUB4	0.073	117 (100.0 %)
	Geo-Mean	84.02	87.45				
	Sensitivity	70.59	76.47				
	Specificity	100.00	100.00				
**Algorithm 2**	Accuracy	79.31	89.66	0.33	R_T1, R_DWI R_SUB3, R_T2 R_SUB4, R_SUB5	0.063	81 (69.2 %)
	Geo-Mean	80.44	90.75				
	Sensitivity	64.71	82.35				
	Specificity	100.00	100.00				
**Algorithm 3**	Accuracy	86.21	89.66	0.22	R_T1, R_DWI R_SUB3, R_T2 R_SUB5, R_SUB4 R_SUB2	0.113	92 (78.6 %)
	Geo-Mean	87.45	90.75				
	Sensitivity	76.47	82.35				
	Specificity	100.00	100.00				

**Table 7:** Results of selection of MRI sequences with perceptual and radiomic features with $\ell_{1}$-norm.

	Performance Measures	Using All Sources (%)	Applying Sources Reduction (%)	Reduction Rate	Relevant Sequences	Required Time (s)	Support Vectors
**Algorithm 1**	Accuracy	76.67	86.67	0.72	P_SUB2, P_SUB5 P_SUB4, P_SUB3 R_SUB3	0.033	117 (100.0 %)
	Geo-Mean	75.51	85.09				
	Sensitivity	82.35	94.12				
	Specificity	69.23	76.92				
**Algorithm 2**	Accuracy	76.67	90.00	0.67	P_SUB2, P_SUB5 P_SUB4, P_SUB3 R_SUB3, R_SUB5	0.039	97 (82.9 %)
	Geo-Mean	75.51	89.24				
	Sensitivity	82.35	94.12				
	Specificity	69.23	84.62				
**Algorithm 3**	Accuracy	76.67	90.00	0.67	P_SUB2, P_SUB5 P_SUB4, P_SUB3 R_SUB3, R_SUB5	0.051	97 (82.9 %)
	Geo-Mean	75.51	89.24				
	Sensitivity	82.35	94.12				
	Specificity	69.23	84.62				

**Table 8:** Comparison of results applying feature selection by Fisher Score technique over the BCI data before and after the source reduction process.

Subject	Feature Selection over All Information Sources				Feature Selection After Reduce Information Sources			
	Accuracy		Minimal Required Features	Reduction Rate (by Sources)	Accuracy		Minimal Required Features	Reduction Rate (by Sources)
	With All Features	Reducing Features			With All Features	Reducing Features		
**Algorithm 1**								
**1**	64.29	85.71	280	0.00	75.00	85.71	362	0.14
**2**	53.33	76.67	667	0.00	76.67	73.33	44	0.59
**3**	60.71	92.86	20	0.68	89.29	92.86	22	0.91
**4**	57.14	75.00	207	0.00	75.00	89.29	23	0.91
**5**	60.00	70.00	130	0.00	63.33	76.67	69	0.82
**6**	63.33	80.00	3	0.95	70.00	80.00	3	0.95
**7**	46.43	67.86	25	0.50	67.86	64.29	1	0.95
**8**	75.00	82.14	52	0.50	85.71	85.71	119	0.82
**9**	71.43	78.57	767	0.00	78.57	75.00	83	0.27
**Average**	61.30 ±8.71	78.76 ±7.70	239.00 ±287.44	0.29 ±0.37	75.71 ±8.23	80.32 ±8.97	80.67 ±112.50	0.71 ±0.31
**Algorithm 2**								
**1**	67.86	85.71	243	0.00	75.00	85.71	300	0.50
**2**	66.67	73.33	166	0.00	66.67	70.00	389	0.36
**3**	71.43	96.43	2	0.91	75.00	96.43	2	0.91
**4**	46.43	75.00	201	0.00	78.57	85.71	20	0.91
**5**	56.67	70.00	281	0.00	66.67	73.33	163	0.77
**6**	70.00	83.33	420	0.00	76.67	80.00	88	0.55
**7**	46.43	67.86	35	0.41	67.86	64.29	32	0.91
**8**	67.86	82.14	62	0.36	82.14	85.71	25	0.86
**9**	71.43	78.57	445	0.00	78.57	75.00	26	0.73
**Average**	62.75 ±10.25	79.15 ±8.88	206.11 ±159.35	0.19 ±0.32	74.13 ±5.73	79.58 ±9.89	116.11 ±140.10	0.72 ±0.21
**Algorithm 3**								
**1**	67.86	85.71	244	0.00	78.57	78.57	97	0.77
**2**	63.33	70.00	167	0.00	63.33	70.00	155	0.05
**3**	64.29	89.29	2	0.91	82.14	96.43	58	0.91
**4**	50.00	67.86	2	0.91	71.43	82.14	7	0.91
**5**	56.67	73.33	281	0.00	66.67	73.33	163	0.77
**6**	66.67	83.33	420	0.00	73.33	80.00	10	0.82
**7**	60.71	71.43	655	0.00	64.29	64.29	32	0.91
**8**	67.86	82.14	5	0.95	82.14	82.14	5	0.95
**9**	71.43	78.57	445	0.00	71.43	78.57	184	0.73
**Average**	63.20 ±6.59	77.96 ±7.62	246.78 ±229.61	0.31 ±0.46	72.59 ±7.17	78.39 ±9.02	79.00 ±72.67	0.76 ±0.28

**Table 9:** Comparison of results applying feature selection by Fisher Score technique over the MRI data before and after the source reduction process.

Algorithm	Feature Selection Over All Information Sources				Feature Selection after Reduce Information Sources			
	Accuracy		Minimal Required Features	Reduction Rate (by Sources)	Accuracy		Minimal Required Features	Reduction Rate (by Sources)
	With All Features	Reducing Features			With All Features	Reducing Features		
**Perceptual Features**								
**1**	73.33	83.33	26	0.11	80.00	80.00	7	0.78
**2**	80.00	90.00	48	0.00	93.33	93.33	26	0.56
**3**	80.00	86.67	23	0.22	86.67	90.00	15	0.67
**Radiomic Features**								
**1**	82.76	96.55	179	0.00	86.21	93.10	171	0.33
**2**	79.31	96.55	173	0.00	89.66	93.10	174	0.33
**3**	86.21	96.55	179	0.00	89.66	93.10	269	0.22
**Perceptual and Radiomic Features**								
**1**	76.67	90.00	302	0.00	86.67	90.00	43	0.72
**2**	76.67	90.00	526	0.00	90.00	90.00	124	0.67
**3**	76.67	90.00	526	0.00	90.00	90.00	125	0.67
